# Supplementary material for: Pentatricopeptide repeat poly(A) binding protein KPAF4 stabilizes mitochondrial mRNAs in Trypanosoma brucei
Source: Nat Commun. 2019 Jan 11;10:146. doi: 10.1038/s41467-018-08137-2 (PMC6329795; doi:10.1038/s41467-018-08137-2)
Supplement: Supplementary file 1 — Supplementary Information [file 41467_2018_8137_MOESM1_ESM.pdf]

Supplementary Information

Pentatricopeptide repeat poly(A) binding protein KPAF4 stabilizes mitochondrial mRNAs in *Trypanosoma brucei*

Mikhail V. Mesitov, Tian Yu, Takuma Suematsu, Francois M. Sement, Liye Zhang, Clinton Yu, Lan Huang and Inna Aphasizheva

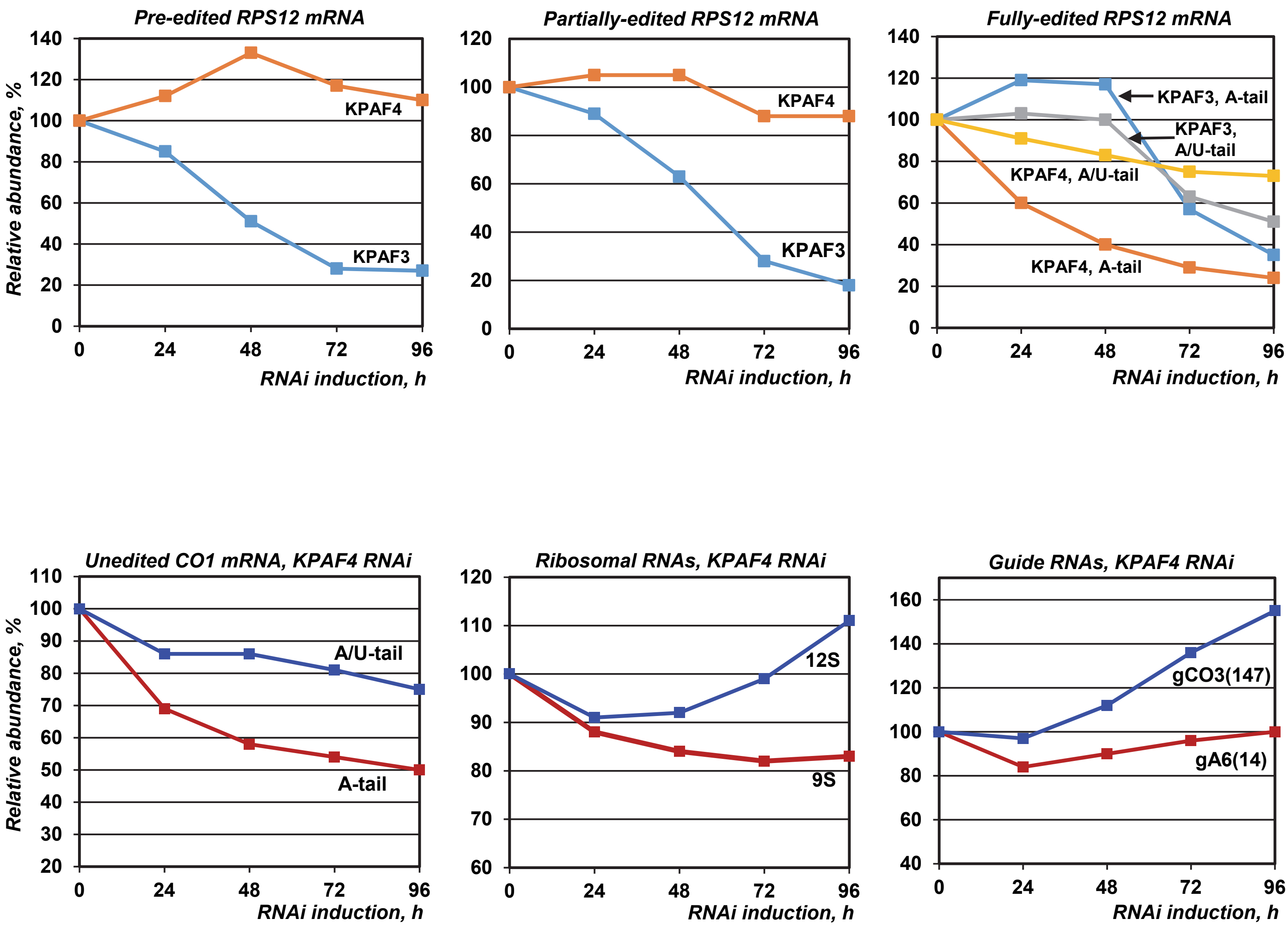

Supplementary Figure 1. Quantitation of northern blotting images shown in Fig. 4. Non-saturated signals were acquired with phosphor storage screen and quantitated vs. indicated nuclear encoded RNAs.

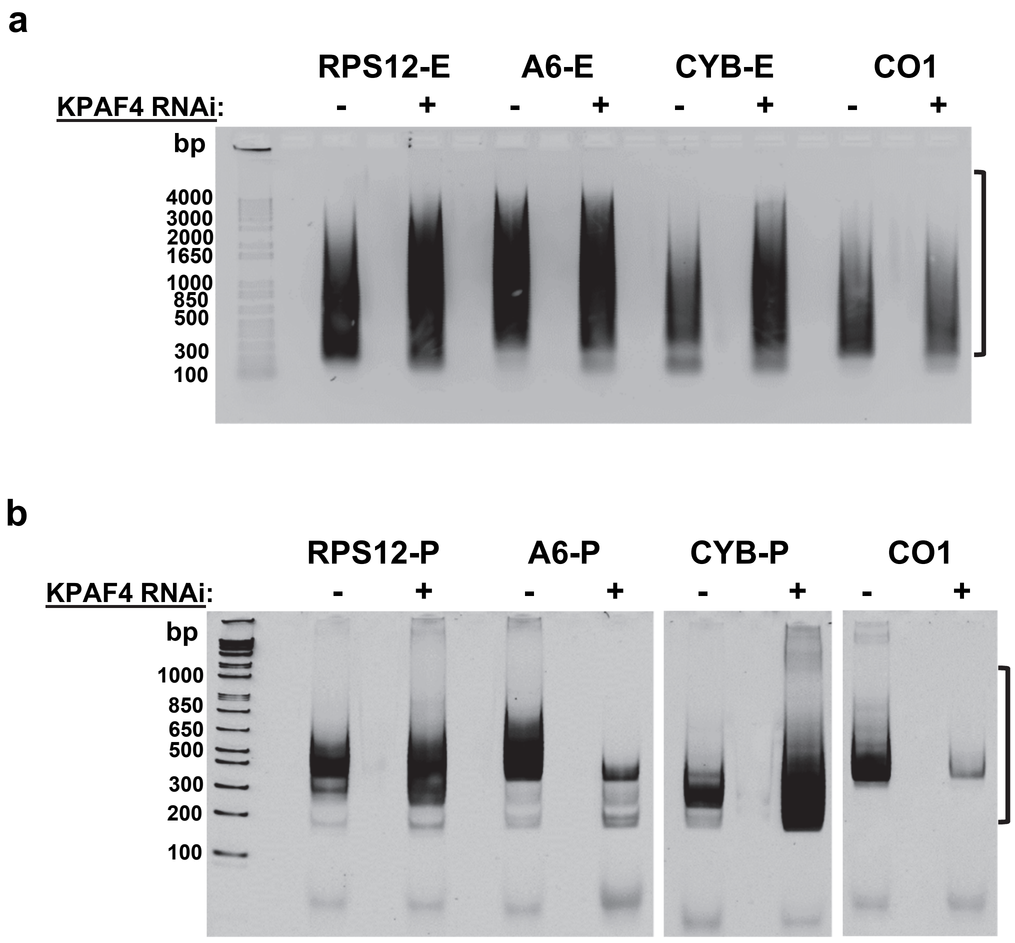

Supplementary Figure 2. Circular RT-PCR libraries for 3' extension analysis. DNA was extracted from regions indicated by brackets. A. SMRT libraries were purified by electrophoresis in 1.2% agarose gel. B. RNA-Seq libraries were purified by electrophoresis in 5% polyacrylamide gel.

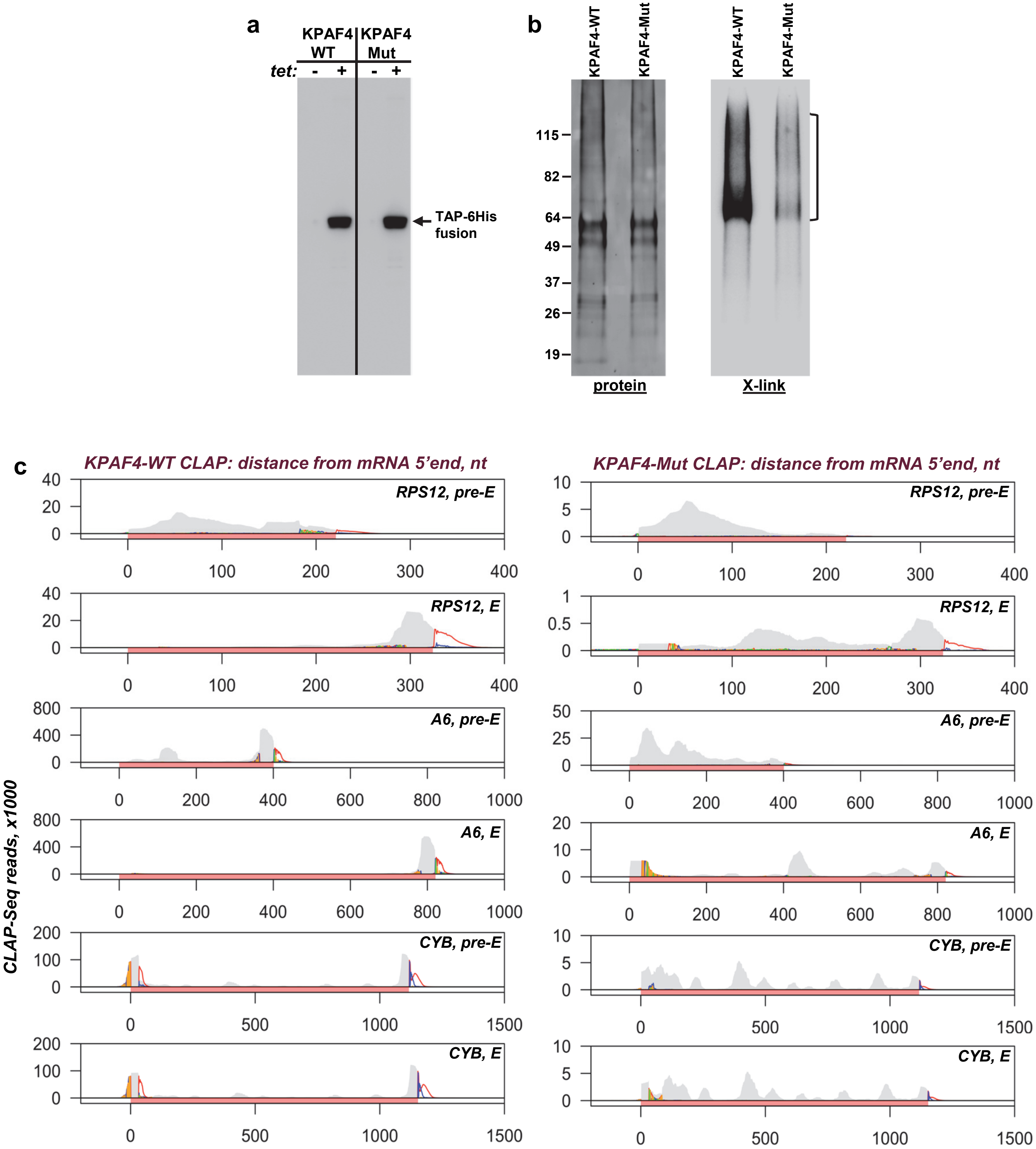

Supplementary Figure 3. KPAF4-Mut expression and *in vivo* binding sites analysis. (a). Inducible KPAF4-WT and KPAF4-Mut expression. Cell lysates were analyzed by western blotting with anti-CBP antibody. (b) Isolation of in vivo KPAF4-RNA crosslinks. Sequenced area is indicated by brackets. (c) Crosslinked fragments were mapped to representative mitochondrial mRNAs.

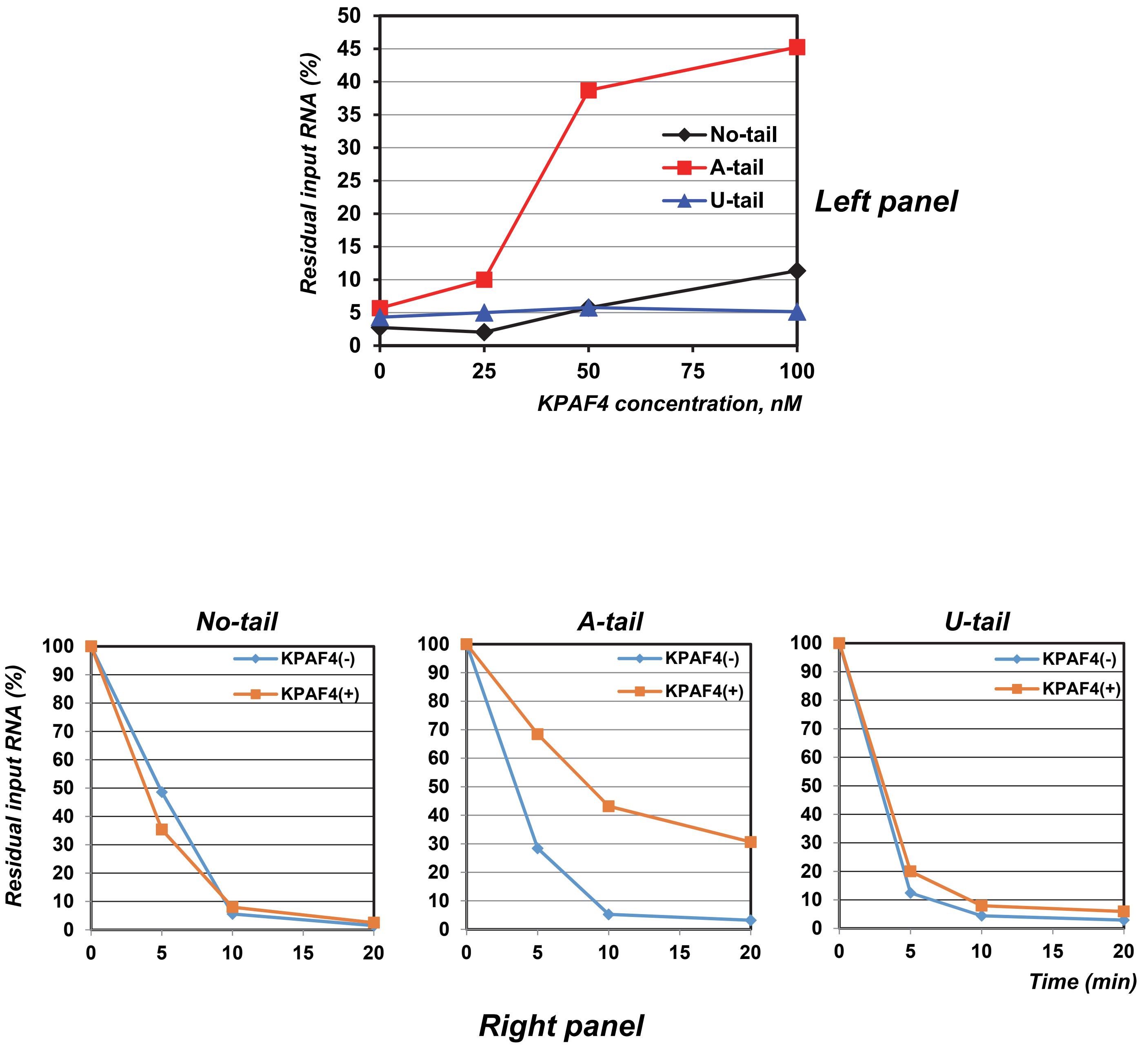

Supplementary Figure 4. Quantitation of input RNA decay in Fig. 7E. Non-saturated signals were acquired with phosphor storage screen and quantitated vs. input RNAs.

Supplementary Methods

Experimental Model

Trypanosoma brucei subsp. *brucei*, strain Lister 427 29-13 (TetR T7RNAP) is a procyclic form cell line that expresses T7 RNA polymerase (T7RNAP) and tetracycline repressor (TetR)[1]. Strain Lister 427 29-13 (TetR T7RNAP) was derived by sequential transfections of the procyclic Lister 427 strain (BEI Resources NR-42010). This cell line was maintained in SDM-79 media supplemented with neomycin, hygromycin and 10% fetal bovine serum at 27°C. For preparative applications, parasites were grown in 1L roller bottles at 8RPM. Protein and RNAi expressing transgenic cell lines were maintained in the same media with phleomycin.

Parasite Maintenance, RNAi, Protein Expression and RNA analysis

Plasmid for RNAi knockdown was generated by amplifying the KPAF4 gene fragment using A715/A716 primer pair, followed by cloning into p2T7-177 vector for tetracycline-inducible expression [2]. Linearized constructs were transfected into a procyclic 29-13 *T. brucei* strain [1]. For inducible protein expression, full-length genes were PCR-amplified from genomic DNA and cloned into pLew-MHTAP vector[3]. Protein expression was induced by adding tetracycline to 1 mg/L and maintained for 72 hours unless otherwise indicated.

Mitochondrial Preparations

Crude mitochondrial fraction. Parasite culture was inoculated at 10^6 cells/ml and grown in 850 ml of SDM-79 media supplemented with 10% FBS and required antibiotics at 27°C in a roller bottle at 8 rpm to 15-20x10^6 cells/ml (60-72 hours). Cells were collected by centrifugation at 3000g for 10 min at 4°C. Cell pellet was resuspended and washed in 50 ml of phosphate buffered saline (PBS) plus 6 mM sucrose. Cells were resuspended in DTE buffer (5 mM Tris-HCl, 1mM EDTA, pH 8.0) to achieve final concentration of 1.2x10^9 cells/ml. During the centrifugation steps, a 50 ml conical tube (rated at g-force of 15,000 or higher) was prepared with pre-calculated volumes of 60% sucrose (12 ml of sucrose per 100 ml of DTE), 150 µl of 1M $MgCl_2$ and 0.2 ml of DNase I solution (5000 U/ml, Sigma, Cat# D5025). Cell suspension in DTE was transferred into 10 ml syringe fitted with 26-gauge needle and push intensely into the prepared sucrose cushion. After gentle mixing, the total volume was brought to 50 ml with STE buffer (20 mM Tris-HCl pH 7.6, 250 mM sucrose, 1 mM EDTA) and lysate was incubated on ice for 15 min. The crude mitochondria pellet was collected by centrifugation at 15,000g for 15 min at 4°C. After a wash step with 50 ml of STE, crude mitochondria pellet was in liquid nitrogen or loaded on density gradient for further purification.

Density gradient isolation. Crude mitochondrial pellet was resuspended in 2 ml of 76% RSTE (76% Renografin (Bracco Diagnostics, Ren°Cal 76 Cat# 086032)) in STE buffer and loaded at the bottom of a 20 – 35% Renografin gradient formed in 20 mM Tris-HCl pH 7.9, 250 mM sucrose, 1 mM EDTA (SW-28 rotor tubes, Beckman). After centrifugation for 2 hours at 24,000 rpm, 4°C, the brown band corresponding to mitochondrial fraction (usually in the middle of the gradient) was harvested by side puncture with 18# needle and transferred in to 50 ml tube. Mitochondrial fraction was diluted 5-fold with cold STE buffer and centrifuged for 20 min at 15,000g, 4°C. After a final wash with 2 ml of STE, pure mitochondria were centrifuged again for 20 mins at 15,000g, 4°C before freezing the pellet in liquid nitrogen.

Glycerol Gradient Fractionation and Native PAGE

Crude mitochondrial pellet (200 mg, wet weight) of, was resuspended in 0.3 ml of Gradient Lysis Buffer (GLB, 30 mM HEPES, pH 7.3, 120 mM KCl, 12 mM $MgCl_2$, 1 mM DTT, 1/10 of Complete Protease Inhibitor, 2 U of Turbo DNase and 1.2% NP40) and incubated on ice for 10 min. The lysate was centrifuged at 21,000g for 15 min at 4°C, and the supernatant was recovered. For RNase treatment, 100 U of RNase I was added to the lysate and incubated on ice for 10 min. The10%-30% glycerol gradient in 25 mM HEPES pH 7.3, 100 mM KCl and 10 mM $MgCl_2$ was prepared for SW41 rotor (Beckman) tubes using Gradient Master (Biocomp). The extract (0.25 ml) was centrifuged for 5 hours at 38,000 rpm in SW41 rotor (Beckman) with slow breaking and 0.56 ml fractions were collected from the top with Gradient Station fractionator (Biocomp Instruments). For native gel, each fraction was supplemented with Coomassie R250 to 0.25% and separated on precast NativePAGE 3-12% Bis-Tris Protein Gels (Thermo Fisher). Proteins were electrotransferred onto nitrocellulose membrane for immunodetection.

Western Blotting and Antibodies

Proteins were separated on precast 8-16% SDS Tris-glycine polyacrylamide gels or NativePAGE 3-12% Bis-Tris Protein Gels (Thermo Fisher) and transferred onto nitrocellulose membrane by electroblotting. Detection was performed with antibodies developed in-house (antigen-purified rabbit polyclonal for KPAP1[4] (1:3000), KPAF1[5] (1:3000), KPAF3[6] (1:5000), GRBC1-2[7] (1:10,000) and mouse monoclonal antibody for RET1[8] (1:100), and anti-CBP (1: 2000, GenScript, Cat. No. A00635), to detect TAP tagged proteins. Quantitative chemiluminescent images were acquired with LAS-4000 imager (GE Healthcare).

Tandem Affinity Purification

Crude mitochondrial pellet (~1 g wet weight) was resuspended in 3 ml of Lysis Buffer (LB, 50 mM Tris-HCl, pH 7.6, 120 mM KCl, 1% NP40, 5 mM $MgCl_2$, 5% glycerol, 1/5 of Complete protease inhibitor tablet (Roche), 20 U TURBO DNase (Thermo Fisher)), and incubated on ice for 15 min. After adding extraction buffer without detergent to 11 ml, mitochondria lysate was sonicated 3 times for 10 seconds at 9W. Extracts were centrifuged at 200,000g for 15 min in SW41 rotor (Beckman), passed through the low-protein binding 0.45 μm filter and supplemented or not with 0.1 mg of RNase A and 2000 U of RNase T1. After incubation on ice for 10 min, extracts were transferred to tubes containing 0.2 ml of IgG Sepharose slurry (GE Life Sciences), pre-washed twice with 10 ml of IgG Binding Buffer (IgG-BB, 20 mM Tris-HCl, pH 7.6, 100 mM KCl, 5 mM $MgCl_2$, 5% glycerol, 0.1% NP40), and incubated for 30 min at 4°C. The suspension was transferred into 2 ml disposable column with bottom filter and washed with 5 full column volumes (CV) of IgG-BB. After extra wash with 2 CV of IgG-BB plus 1 mM DTT, the column was closed, and contents incubated with 150 U of AcTEV protease and 1/20 of Complete inhibitor tablet in 1.5 ml of IgG-BB plus 1 mM DTT. After closing the upper end with Parafilm, the column was incubated for 16 hours at 4 °C on a Nutator platform. The column was drained into 15 ml plastic tube containing 0.2 ml of pre-washed Calmodulin Affinity Resin (Agilent Technologies) with calmodulin binding buffer (CBB, 20 mM Tris-HCl, pH 7.6, 100 mM KCl, 2 mM $CaCl_2$, 1 mM MgAc, 0.1% NP40, 10 mM 2-mercaptoethanol, 1 mM imidazole, 5% glycerol). The IgG column was rinsed 4 times with 1 ml of CBB, $CaCl_2$ was added to 1 mM and the suspension was incubated for 1 hour on Nutator at 4°C. The suspension was transferred into 2-ml disposable column and washed with 3 full CV of CBB and protein was eluted with 0.6 ml of Calmodulin Elution Buffer

(CEB, 20 mM Tris-HCl, pH 7.6, 100 mM KCl, 2 mM EDTA, 3 mM EGTA, 0.1% NP-40, 10 mM 2-mercaptoethanol, 1 mM imidazole, 5% glycerol).

Rapid Affinity Purification

Crude mitochondrial pellet (~1 g wet weight) was frozen in liquid nitrogen, powdered with CryoMill (Retsch) and stored at -80°C. All procedures were done at 4°C. Extract was prepared by resuspending frozen powder in 3 ml of pre-warmed lysis buffer (50 mM Tris-HCl pH7.6, 60 mM KCl, 12 mM $MgCl_2$, 1% NP-40, 5% glycerol) containing 0.3 ml of Compete protease inhibitor solution and incubated on ice for 10 min with 0.1 mg of RNase A and 20 U of TURBO DNase (Ambion). Extract was diluted with Extraction buffer (50 mM Tris-HCl (pH7.6), 60 mM KCl, 12 mM $MgCl_2$, 5% glycerol) up to 11 ml and centrifuged at 200,000 x g for 30 min. Supernatant was filtered through 0.45 µm low protein binding filter into 15 ml conical tube with 20 mg of IgG-coated Dynabeads magnetic beads. The extract was incubated on Nutator for 20 min. Beads were collected on DynaMag-15 magnet stand (Life Technologies), rinsed twice with 6 ml of wash buffer (20 mM Tris-HCl (pH 7.6), 500 mM KCl, 12 mM $MgCl_2$, 0.1% NP-40, 5% glycerol) and washed 3 times with 10 ml of wash buffer for 5 min. Beads were transferred into 1.5 ml low protein binding tube and washed twice with 1 ml of wash buffer using the magnetic holder (Life Technologies). To elute TAP-tagged proteins, beads were incubated with 0.3 ml of TEV digestion buffer (20 mM Tris-HCl (pH 8.0), 0.5 mM EDTA, 1 mM dithiothreitol (DTT)) containing 20 U of TEV protease and 6 µl of Compete protease inhibitor solution for overnight on Nutator at 4°C. Beads were pelleted using the magnetic holder and supernatant was collected as eluate. Protein concentration was determined Sypro Ruby staining of SDS gel and quantitating the KPAF4 band vs. known amounts of marker proteins.

Purification of Enzymes for Reconstitution Studies

RNA editing TUTase (RET1)[9] and Kinetoplast poly(A) polymerase[4] were purified from *E. coli* to apparent homogeneity, as described. An MPsome subunit, DSS1 3′-5′ exonuclease was isolated by tandem affinity purification from crude mitochondrial fraction as described[8].

Electrophoretic Mobility Shift Assay (EMSA)

Edited RPS12 mRNA fragments with A-tail, U-tail or no tail as RNA substrates (described below for *in vitro* enzymatic assay) were prepared by *in vitro* transcription. KPAF4 RNA-binding activity was tested in 10 µl reactions containing 20 mM Tris–HCl pH 7.6, 10 mM $MgCl_2$, 1 mM DTT, 50 nM of 5′-labeled ssRNA substrate, and 5, 15, 50 and 150 nM of KPAF4 purified by rapid pulldown with IgG beads. The reaction mixtures were incubated at 30°C for 15 min and immediately placed on ice. In each reaction 3.4 µl of was added and 10 µl of the mixture was loaded on. Each reaction was supplemented with Native PAGE 4x Sample Buffer (Thermo Fisher Scientific) to 1x, and 10 µl of the mixtures was separated on precast NativePAGE 3-12% Bis-Tris Protein Gels (Thermo Fisher Scientific) at 2 W for 1 hour. Gels were dried and exposed to storage phosphor screens. Phosphor images were acquired with Typhoon FLA 7000 (GE Healthcare).

In vitro RNA Adenylation and Uridylation Assays

Reconstitutions were set up in 50 µl reactions containing 20 mM Tris–HCl pH 7.6, 10 mM $MgCl_2$, 1 mM DTT, 50 nM of 5′-labeled ssRNA substrate, 0.1 mM ATP, 0.1 mM of UTP, 100 nM of KPAF4 purified by rapid pulldown (RNase treated), 20 nM of RET1 and 100 nM of KPAP1. The reaction mixture except for RET1 and KPAP1 was pre-incubated at 30°C for 10 min, and the

reaction was started with addition of both enzymes. Aliquots (10 µl) were withdrawn after 5, 10 and 20 min, mixed with 15 µl of Stop Solution (95% formamide, 10 mM EDTA, 0.05% Xylene cyanol and 0.05% Bromophenol blue) and kept on ice until the last time point completed. All aliquots were heated for 3 min at 65°C, incubated on ice for 5 min and separated on 10% acrylamide/bis-acrylamide (19:1)/8M urea denaturing gel. Gels were dried and exposed to storage phosphor screens. Phosphor images were acquired with Typhoon FLA 7000 (GE Healthcare).

In vitro RNA Degradation Assay

MPsome assays were carried out in 20 µl reaction containing 50 mM Tris-HCl (pH 8.0), 1 mM DTT, 2 U/µl RNaseOut (Thermo Fisher Scientific), 0.1 mM $MgCl_2$, 20,000 cpm of 5′-labeled ssRNA, 2 µl of TAP-purified DSS1 fraction and 50 nM of KPAF4 purified by rapid affinity pulldown. The reaction mixture was pre-incubated for 20 min at 30°C, and the reaction was started with addition of DSS1 fraction. Aliquots (5 µl) were withdrawn after 5, 10 and 20 min, mixed with 5 µl of Stop Solution (95% formamide, 10 mM EDTA, 0.05% Xylene cyanol and 0.05% Bromophenol blue) and kept on ice until the last time point completed. All aliquots were heated for 3 min at 65°C, incubated on ice for 5 min and separated on 10% acrylamide/bis-acrylamide (19:1)/8M urea denaturing gel. To measure the length of products, the same RNA substrates were digested by guanine-specific RNase T1 and alkaline-hydrolyzed. RNase T1 digestion was carried out in 10 µl reaction containing 3 mM sodium citrate (pH 4.5), 0.1 mM EDTA, 0.8 M urea, 0.5 µg/µl yeast tRNA mixture (Ambion), 0.02 U/µl RNase T1 (Ambion), and 10,000 cpm of labeled ssRNA. The reaction was incubated for 2 min 30 s at 55 °C and kept on ice. The equal volume of Stop Solution was added to the reaction prior to loading. Partial alkaline-hydrolysis was carried out in 10 µl reaction containing 100 mM sodium carbonate (pH 9.0) and 10,000 cpm of labeled ssRNA. The reaction was incubated for 2 min 30 s at 95°C and kept on ice. The equal volume of Stop Solution was added to the reaction prior to loading. Gels were dried and exposed to storage phosphor screens. Phosphor images were acquired with Typhoon FLA 7000 (GE Healthcare).

BioID

The protocol was adapted from[10]. Approximately 1 g of crude mitochondrial fraction (wet weight) was resuspended in 3 ml of extraction buffer (EB, 50 mM Tris-HCl, pH 7.6. 150 mM NaCl, 1% NP40, 5 mM $MgCl_2$) with 200 µl of 20X Complete inhibitor EDTA free, and transferred into 15 ml tube. Turbo DNase (5U) and RNase A (0.1 mg) were added and incubated on ice for 15 min. EBE buffer (50 mM Tris-HCl, pH 7.6. 150 mM NaCl, 10 mM EDTA) was added to final 11 ml and the extract was sonicated 3 x 15 sec with microtip at 12 W. The extract was cleated at 40,000 RPM for 20 min in SW41 rotor. Supernatant was filtered through 0.45 µ low protein binding filter and incubated overnight with 0.1 ml of Streptavidin Dynabeads (Thermo Fisher). Beads were washed at room temperature in 2 ml tubes with 1 ml of buffer for 10 min as follows: 2x with 20 mM Tris-HCl, pH 7.6, 150 mM NaCl, 1 mM EDTA, 0.1 % NP-40 - 50 ml; 2x with 2% SDS; 2x with 20 mM Tris pH 7.5, 500 mM NaCl, 0.1% deoxycholate, 1% Triton X-100, 1 mM EDTA; 2x with 20 mM Tris pH 8, 250 mM LiCl, 0.5% NP-40, 0.5% deoxycholate, 1 mM EDTA; 2x with 50 mM Tris, pH 7.4, and 50 mM NaCl, 5 mM DTT; 2x with 50 mM NH4HCO3, pH 7.5. Beads were transferred into a fresh low protein biding tube and incubated overnight at 37 ^{0}Cwith 50 ng of trypsin in 0.1 ml of 50 mM NH4HCO3, pH 7.5. Peptides was purified by applying the solute onto Vivapure spin column (Sartorius) and proceeding with manufacturer’s protocol.

Protein Identification by LC-MS/MS

Affinity-purified complexes were precipitated by addition of trichloroacetic acid and deoxycholate to 20% and 0.1%, respectively, washed three times with ice-cold acetone, and digested with LysC peptidase in 8M urea (1:50 ratio) for 4 hours at 37 ºC. Reaction was diluted five-fold with 50 mM Na-bicarbonate pH 7.5 and further digested with trypsin (1:100 ratio) for 16 hours. Peptides were purified on Vivapure spin columns (Sartorius). LC-MS/MS was carried out by nanoflow reversed phase liquid chromatography (RPLC) (Eksigent, CA) coupled on-line to a Linear Ion Trap (LTQ)-Orbitrap mass spectrometer (Thermo-Electron Corp). The LC analysis was performed using a capillary column (100 µm ID x 150 mm) with Polaris C18-A resin (Varian Inc., CA). The peptides were eluted using a linear gradient of 2% to 35% B in 85 min at a flow of 300 nL/min (solvent A: 100% H2O, 0.1% formic acid; solvent B: 100 % acetonitrile, 0.1% formic acid). A cycle of full FT scan mass spectrum (m/z 350-1800, resolution of 60,000 at m/z 400) followed by ten data-dependent MS/MS spectra acquired in the linear ion trap with normalized collision energy (setting of 35%). Target ions already selected for MS/MS were dynamically excluded for 30 s. Monoisotopic masses of parent ions and corresponding fragment ions, parent ion charge states and ion intensities from the tandem mass spectra (MS/MS) were obtained by using in-house software with Raw_Extract script from Xcalibur v2.4. Following automated data extraction, resultant peak lists for each LC-MS/MS experiment were submitted to the development version of Protein Prospector (UCSF) for database searching similarly as described [11]. Each project was searched against a normal form concatenated with the random form of the *T. brucei* database (http://tritrypdb.org/tritrypdb/). Trypsin was set as the enzyme with a maximum of two missed cleavage sites. The mass tolerance for parent ion was set as ± 20 ppm, whereas ± 0.6 Da tolerance was chosen for the fragment ions. Chemical modifications such as protein N-terminal acetylation, methionine oxidation, N-terminal pyroglutamine, and deamidation of asparagine were selected as variable modifications during database search. The Search Compare program in Protein Prospector was used for summarization, validation and comparison of results. Protein identification is based on at least three unique peptides with expectation value ≤ 0.05.

RNA Isolation

Cell culture equivalent of $25x10^7$ cells was collected by centrifugation at 3000 g for 10 min at 4°C, washed with 50 ml of PBS, collected likewise, transferred to 2 ml tube, and re-pelleted at 3000 g for 5 min at 4°C. Cell pellets were resuspended in 0.8 ml of cold Solution D (4M guanidine isothiocyanate, 25 mM sodium citrate, pH 7.0, 0.5% sarcosyl, 0.1 M 2-mercaptoethanol) and supplemented with 0.1 ml of 2 M sodium acetate (pH 4.0) and 0.9 ml of water-saturated phenol, and gently mixed. After addition of 0.3 ml of chloroform/isoamyl alcohol (49:1), lysates were incubated for 10 min on Nutator at 4°C. Phases were separated by centrifugation at 21,000 x g for 10 min, and the supernatant was transferred into a Phase Lock Gel Heavy 2-ml tube (5Prime) and extracted vigorously by vortexing for 1 min with 0.8 ml of chloroform/isoamyl alcohol. Supernatant was transferred into a fresh tube and RNA was precipitated with 1 ml of isopropanol at -20°C for 1 hour. RNA was collected by centrifugation at 21,000 x g for 15 min and washed with 80% ethanol. RNA pellet was dissolved in 0.2 ml of water and re-precipitated with sodium acetate/ethanol by standard technique. For Northern blotting, 50 µg of RNA was treated with 5U of RNase-free DNase I (New England Biolabs) in 0.1 ml of manufacturer-supplied buffer for 30 min at 37°C. RNA was extracted with equal volume of phenol (pH 5), and phenol-chloroform, and precipitated with ethanol. For quantitative RT-PCR, a Turbo DNase digestion was performed for 30 min at 37°C and RNA was purified with RNeasy MiniElute Kit (Qiagen) as recommended by

the manufacturer. For RNA circularization, 20 μg was treated with 20 U of DNase I, Amplification Grade (Thermo Fisher Scientific) for 15 min at 25°C. RNA was purified with RNA Clean and Concentrator-25 kit (Zymo Research) with exclusion of small RNAs (< 200 nt), following manufacturer's instruction.

Northern Blotting

Northern blotting experiments were performed according to published protocol [12]. Total DNase-treated RNA (8 -12 μg) was separated on 20 cm-long 5% (mRNA and rRNA detection) or 10% (tRNA and gRNA detection) acrylamide/bis-acrylamide (19:1)/8M urea gel. RNAs were transferred to a BrightStar®-Plus membrane (Thermo Fisher Cat# AM10104) by tank transfer at 100 V for 2 hours at 4°C. Alternatively, total RNA was separated on 20 cm-long 1.7% agarose/formaldehyde gels (CO1, ND1, A6 and CYB mRNA detection). After electrophoresis, RNA was transferred to a BrightStar®-Plus membrane (Thermo Fisher Cat# AM10104) using vacuum blotting system at 50 mbar for at least 4 hours or overnight. After transfer, RNA was cross-linked to membrane by exposition to UV light at 120 mJ/cm2 using CX-2000 UV Crosslinker (UVP). Oligo probes were labeled by incubating 10 pmol of oligonucleotide with T4 polynucleotide kinase (Ambion) in the presence of 6.5 μl of [γ-32P] ATP (6000 μCi/ml) (Perkin Elmer) for 30 min at 37°C. Labeled oligoprobe was purified on G-25 Sephadex column (GE Life Sciences). For single-stranded DNA probe, the template was amplified with oligos used for quantitative RT-PCR. DNA recovered from a single 25 μl qRT-PCR reaction was typically sufficient for 20 single-strand probe preparations by asymmetric two-step PCR: 10 pmol of 5-radiolabeled antisense oligonucleotide in a standard PCR reaction (98°C, 30 s; 98°C, 10 s; 50°C, 30 s; 45 cycles) with Phusion Hot Start II DNA Polymerase (Thermo Fisher Scientific). The probe was purified on G25 Sephadex spin column (GE Life Sciences). For the detection of antisense mRNA, the radiolabeled ssDNA probe was produced using the sense oligonucleotide (instead of antisense) in the PCR reaction. Hybridization with radiolabeled probes was performed overnight using ULTRAhyb ™ Ultrasensitive Hybridization buffer (ThermoFisher Cat# AM8670) or ULTRAhyb ™-Oligo Hybridization buffer (ThermoFisher Cat# AM8663). Oligonucleotides and ssDNA PCR products were hybridized overnight at 40°C and 42°C, respectively. Membranes were washed 3 times at the hybridization temperature with 15 ml of 2x SSPE 0.5% SDS or 4x SSPE 0.5% SDS for PCR probes and oligonucleotides, respectively. Phosphor images were acquired with Typhoon FLA 7000 (GE Healthcare).

Quantitative RT-PCR

cDNA was synthesized from 2 μg of TURBO DNase-treated, column purified total RNA in 0.1 ml reaction with TagMan Reverse Transcription Reagents (N808-0234, Applied Biosystems) as recommended by the manufacturer. Primer pairs were pre-mixed at 1.5 μM final concentration of each in water. In a 1.5 ml tube, 8 μl of cDNA was mixed with 18 μl of primer mix. Power SYBR Green Master Mix was added to 50 μl. Triplicate aliquots of 16 μl were distributed into 96 well plate (951022043, Eppendorf). After sealing the plate with a film (951023060, Eppendorf), PCR reactions were performed in Eppendorf Realplex 2S cycler as follows: 95°C, 10 min; 95°C (15 s), 60°C (1 min, measure point), 45 cycles.

RNA Circularization and Reverse Transcription

For cRT-PCR experiments, KPAF4 RNAi was induced for 72 hours. DNase I-treated and column-purified total RNA (10 μg) was circularized in 50 μl reaction with 30 U of T4 RNA ligase 1 (New

England Biolabs) in the presence of 1 mM ATP, 5 mM DTT, 40 U of RNase OUT (Thermo Fisher Scientific) and 10% of PEG 8000 overnight at 14°C, followed by incubation for 30 min at 37°C. Remaining linear RNA was digested with 0.1 U/µl of RNase R (Epicenter) for 10 min at 37°C. Circular RNA was phenol/chloroform extracted and precipitated with ethanol. Complementary DNA was synthesized from 2.5 µg of purified circular RNA in 20 µl reaction with 20 U/µl of SuperScript III Reverse Transcriptase (Thermo Fisher Scientific) using gene-specific primer (1 µM) as recommended by the manufacturer. RNA was digested with 1 U of RNase H (Thermo Fisher Scientific), 2.5 U of RNAse T1 and 1 µg of RNase A in RNace-IT RNase cocktail (Agilent) for 20 min at 37°C.

Library Preparation for Single Molecule Real-time (SMRT) Sequencing

The region spanning mRNA 5′ end, tail and 3′ end was amplified in a 100 µl PCR reaction in the presence of 500 pmol of each transcript-specific primer using 4 µl of cDNA as a template and 2 U of Phusion Hot Start II DNA Polymerase (Thermo Fisher Scientific) according to manufacturer recommendations. After an initial denaturation step of 30 s at 98°C, the PCR reactions underwent 30 PCR cycles (98°C 10 s, 55°C 30 s, 68°C 30 s) and a final elongation step of 5 min at 68°C. After purification of the PCR products on a ZYMO DNA clean and concentrator-5 column and elution in 12 µl, the PCR products were resolved on a 1.2% agarose gel. After staining the gel with SYBR Green 1, the area of interest (~0.2 bp – 4 kb) was excised under blue light and PCR products were extracted using QIAquick Gel Extraction Kit (Qiagen) following manufacturer's instruction. Purified DNA libraries were concentrated by ethanol precipitation in the presence of 5 µg of glycogen and dissolved in 10 µl of 10 mM Tris-HCl, pH 8.0. Size distribution of the libraries was analyzed by 2100 Bioanalyzer (Agilent). Sequencing of the libraries was performed on PacBio RS II system.

Library Preparation for Illumina MiSeq Sequencing

For MiSeq sequencing the libraries were generated in two sequential PCR reactions. First PCR was performed in a 100 µl reaction in the presence of 500 pmol of each transcript-specific primer using 5 µl of the cDNA synthesis reaction and 2 U of Phusion Hot Start II DNA Polymerase (Thermo Fisher Scientific) according to manufacturer recommendations. After an initial denaturation step of 30 s at 98°C, the PCR reactions underwent five three-step PCR cycles (98°C 10 s, 55°C 30 s, 72°C 20 s) and five additional two-step PCR cycles with higher annealing temperature (98°C 10 s, 72°C 30 s) followed by final elongation step of 1 min at 72°C. PCR products were purified with DNA Clean and Concentrator-5 kit (Zymo Research) and eluted in 20 µl of 10 mM Tris-HCl, pH 8.0. Second PCR was performed in 100 µl reaction with the presence of 600 pmol of each Illumina universal forward primer and Illumina indexed reverse primers, using 10 µl of the purified first PCR reaction and Phusion Hot Start II DNA Polymerase (Thermo Fisher Scientific). After an initial denaturation step of 30s at 98°C, the PCR reactions underwent 16 PCR cycles (98°C 10 s, 60°C 30 s, 72°C 20 s) and a final elongation step of 1 min at 72°C. After purification of the PCR products on a ZYMO DNA clean and concentrator-5 column and elution in 12 µl, PCR products were resolved on a 6% acrylamide/TBE gel. After staining the gel with SYBR Green 1, the area of interest was excised under blue light. PCR products were extracted with 5 volumes of extraction buffer (0.5 M ammonium acetate, 0.1% SDS and 1 mM EDTA) for overnight at room temperature on a Nutator. The supernatant was concentrated 5 - 6 times with 2-butanol, DNA was extracted with phenol/chloroform, precipitated with ethanol and cleaned-up using DNA Clean and Concentrator-5 kit (Zymo Research). Size distribution of the libraries was

analyzed by 2100 Bioanalyzer (Agilent). Deep sequencing of the libraries was carried out on Illumina MiSeq platform in a single-end 300 bp mode.

Crosslinking-Two Step Affinity Purification and Sequencing (CLAP-Seq)

T. brucei cells were seeded at 10^6 cells/ml in 850 ml of SDM-79 medium supplemented with 10% FBS. Expression of TAP-tagged protein was induced with 2 µg/ml of tetracycline, and the cells were grown to ~$20x10^6$ cells/ml for ~72 hours post-induction. Cells were pelleted at 3000g for 15 min, washed with 50 ml of ice-cold PBS with 6 mM sucrose and resuspended in 32 ml ice-cold PBS with 6 mM sucrose. Half of suspension volume was distributed equally into 4 pre-chilled cover plates from 10 cm Petri dishes. The remaining half was kept on ice. Petri dishes were placed on 6 cm-tall cold blocks and irradiated three times at 400 mJ/cm2 in CX-2000 UV Crosslinker (UVP) with gentle mixing between UV cycles. Cells were transferred into 50 ml tube and 30 ml of PBS was used to collect the remaining material from Petri dishes. Crosslinked and control cells were collected at 3000g for 10 min and frozen with liquid nitrogen. UV-irradiated and mock-treated cell pellets were resuspended in 3 ml of Extraction Buffer (EB, 50 mM Tris-HCl, pH 7.6. 150 mM NaCl, 2 mM MCl_2, 1%NP40, 1/20 of Complete Protease Inhibitor tablet without EDTA, 40U of TURBO DNase) per gram of cells (wet weight) and incubated on ice for 10 min. Cells were diluted with 50 mM Tris-HCl, pH 7.6. 150 mM NaCl, 2 mM MCl_2, 1%NP40 buffer to 11 ml and sonicated 3 times for 20 s at 12W with intermediate incubations on ice. The extracts were centrifuged at 40,000 rpm for 20 min in SW41 rotor. The supernatant was filtered through 0.22 µm low protein binding filter, EDTA was added to 5 mM. RNA was partially digested with 40 U/ml of RNase I for 5 min at 37°C. IgG-Fast Flow Sepharose (350 µl of slurry) (GE Life Sciences) was added per tube and incubated on Nutator for 30 min at 4°C. The mixture was transfered to 1 ml plastic column (Pierce), the buffer was drained, and the loading was repeated one more time with the flow through. Each column was sequentially washed 3 times with 10 ml of IgG-Wash buffer (WB, 20 mM Tris-HCl pH 7.6, 150 mM NaCl, 0.1%NP40), 2 times with 10 ml of IgG High Salt Wash buffer (HS, 20 mM Tris-HCl pH 7.6, 500 mM NaCl, 0.1%NP40) and 2 times with 10 ml of WB. To elute the protein-RNA crosslinks 400 µl of Ni-Binding Buffer (6M Guanidium Chloride, PBS pH7.6, 10 mM Imidazole) was added and the resin was incubated for 5 min at room temperature. Elution step was repeated three times collecting the eluate into the same 2 ml tube. His-Pur Ni-NTA magnetic beads (0.5 mg) (Thermo Fisher Scientific) were added per tube and incubated in Thermomixer (Eppendorf) at 1000 rpm for overnight at room temperature. Next day the beads were washed three times with 1 ml of Ni-Binding Buffer for 5 min at 1000 rpm at 25°C followed by 3 times washing with WB and 2 times washing with CIAP buffer (40 mM Tris pH 8.0). After the last wash, the beds were in 50 µl of CIAP buffer with 25 U of CIAP (Thermo Fisher Scientific) for 20 min at 37°C at 800 RPM. After three washes with 1 ml of PNK buffer (40 mM Tris-HCl, pH 7.5, 10 mM $MgCl_2$, 0.1% NP40) for 5 min at 37°C at 800 RPM, the beds were incubated in 50 µl volume containing 10 µl of 5x T4 kinase buffer (Thermo Fisher Scientific), 20U of T4 Polynucleotide kinase (Thermo Fisher Scientific), 1 µl (γ-^{32}P)ATP (Perkin Elmer), 40 U of RNaseOut (Thermo Fisher Scientific) and 1 µl of freshly made 0.5M β- mercaptoethanol. The mixture was incubated for 10 min at 37° at 800 RPM. After 10 min of the incubation, rATP (Promega) was added to a final concentration of 0.2 mM, and the mixture was incubated for additional 30 min. After two washes with 1 ml of Ni-Binding Buffer and two with PNK, ribonucleoprotein complexes were eluted with 20 µl of 1xLDS-MOPS loading buffer with 200 mM Imidozole by incubating for 10 min at room temperature, 1000 rpm. Eluate was collected and incubated for 3 min at 60°C after addition of 1 µl of 1M DTT, 1000 rpm. After centrifugation of

the supernatant for 4 min at 21,000g, eluates were resolved on a 4%-12% NuPAGE gel (Thermo Fisher Scientific). Proteins were transferred to nitrocellulose membrane in 1xMOPS buffer as recommended by manufacturer and exposed to phosphor storage screen. The membrane was stained with Sypro Ruby protein blot stain (Thermo Fisher) for 2-3 min and de-stained in water. The area of interest was cut out under blue light into strips and incubated in 200 µl of PK buffer (100 mM Tris-HCl, pH 7.5, 50 mM NaCl, 10 mM EDTA) with 4 mg/ml of proteinase K at 37 °C for 20 min at 1000 rpm. The mixture was supplemented with 200 µl of 8M urea in 1x PK buffer and incubated for 20 more minutes. RNA was purified by adding 200 µl of water-saturated phenol and 130 µl of chloroform and incubated for 20 min at 37°C at 1000 rpm. After centrifugation at 21,000g for 5 min at room temperature, the upper aqueous phase was transferred to a new tube and RNA was re-extracted with 0.2 ml of chloroform in the presence of .40 µl of 3M Sodium Acetate (pH 5.2) and 5 µg of glycogen by incubation for 5 min at 37°C at 1200 rpm. Extraction was repeated one more time with 0.1 ml of chloroform for 5 min at 37°C at 1200 rpm. After final centrifugation the RNA was precipitated with 1 ml of ethanol:isopropanol (1:1) mixture. The pellet was washed with 80 % ethanol and dissolve in 20 µl of RNase/DNase free water. RNA was precipitated second time with 2.5 µl of 3M Sodium Acetate (pH 5.2) and 90 µl of ethanol for overnight at -20°C. Pelleted and washed RNA was dissolved in 5 µl of water and used for cDNA synthesis and library preparation with NEB Next Multiplex Small RNA Library Prep Set for Illumina (New England BioLabs) according to manufacturer's protocol. DNA libraries were amplified in 15 cycles PCR reaction. After purification of the PCR products on a ZYMO DNA clean and concentrator-5 column and elution in 12 µl, the PCR products were resolved on a 6% acrylamide/TBE gel. After staining the gel with SYBR Green 1, the area of interest was excised under blue light. PCR products were extracted with 5 volumes of extraction buffer (0.5 M ammonium acetate, 0.1% SDS and 1 mM EDTA) for overnight at room temperature on a Nutator. The supernatant was concentrated 5 - 6 times with 2-butanol, DNA was extracted with phenol/chloroform, precipitated with ethanol and cleaned-up using DNA Clean and Concentrator-5 kit (Zymo Research). Size distribution of the libraries was analyzed by 2100 Bioanalyzer (Agilent). Deep sequencing of the libraries was carried out on Illumina HiSeq platform in a paired-end 150 bp mode.

Quantification and Statistical Analysis

CLAP-Seq

Raw KPAF4 CLAP-Seq and MERS1 CLAP-Seq data was obtained in the form of pair-end reads in FastQ format. For both datasets, raw read pairs were subjected to adapter trimming by cutadapt (v1.14, pypi.python.org/pypi/cutadapt/), and the minimum length of processed reads was set to 20 nt. Adapter-free read pairs were merged via PEAR (v0.9.8, sco.h-its.org/exelixis/web/software/pear/) [13], with minimum assembly length set to 15 nt. After pre-processing, the clean reads of KPAF4 CLAP-Seq and MERS1 CLAP-Seq were mapped to the *T. brucei* nuclear genome and matched reads were filtered out. The reference nuclear genome was retrieved from TriTrypDB (Release 33, tritrypdb.org/tritrypdb/). The remaining KPAF4 CLIP-Seq reads were mapped to the full maxicircle sequence (Genbank ID: M94286.1) and to edited mRNA sequences. The read mapping was performed using Bowtie2 (v2.3.2, http://bowtie-bio.sourceforge.net/bowtie2/index.shtml) with default parameters. In-house Perl script "sam2covR.pl" was used to read the SAM file from Bowtie2 and calculate the number of mapped reads per nucleotide for both the major and minor strand of the maxicircle. The output data was subsequently used to visualize read mapping coverage using in-house R-script by plotting the

maxicircle coordinates on the X-axis and the corresponding read counts on the Y-axis. To identify reads originated from mRNA 3′ ends including tails we performed local alignment using BWA local aligner (bio-bwa.sourceforge.net) with default settings. In-house Perl script "sam2maskedBaseFreq.pl" was used to interpret the CIGAR string of the SAM files produced by BWA and calculate both the read count for each position and the nucleotide frequencies of the unmapped part of the reads. In-house R-script were used to visualize the read coverage as well as the unmapped nucleotide frequencies. After processing in the same pipeline, a negligible number of reads was recovered from mock-irradiated samples.

Sequencing of mRNA 3′ ends

Raw Tail-Seq sequencing data was obtained in the form of FastQ files. To extract mRNA tails, we used cutadapt (v1.14, pypi.python.org/pypi/cutadapt/) to remove 5′ and 3′ flanking regions that surrounded the tail sequences. To compensate for the relatively high error rate of PacBio SMRT sequencing, the error tolerance rate of cutadapt was set to 0.15. To remove false tails of artificial origin, we filtered out tails that have abnormally low AT percentage (less than 87%) using in-house Perl script "clean_tails.pl". By removing the flanking regions, the process would also separate tail sequences by their originating mRNAs at the same time. For tails from each mRNA, in-house R-script was used to visualize the tail length distribution. An in-house Perl script "baseCtByPos.pl" was used to calculate the nucleotide frequency of tail sequences from each mRNA, and in-house R-script was used to visualize it.

Supplementary References

1. Wirtz, E., Leal, S., Ochatt, C. & Cross, G.A. A tightly regulated inducible expression system for conditional gene knock-outs and dominant-negative genetics in Trypanosoma brucei. *Mol. Biochem. Parasitol* **99**, 89-101 (1999).
2. Wickstead, B., Ersfeld, K. & Gull, K. Targeting of a tetracycline-inducible expression system to the transcriptionally silent minichromosomes of Trypanosoma brucei. *Mol. Biochem. Parasitol* **125**, 211-216 (2002).
3. Jensen, B.C. et al. Characterization of protein kinase CK2 from Trypanosoma brucei. *Mol Biochem Parasitol* **151**, 28-40 (2007).
4. Etheridge, R.D., Aphasizheva, I., Gershon, P.D. & Aphasizhev, R. 3' adenylation determines mRNA abundance and monitors completion of RNA editing in T. brucei mitochondria. *EMBO J* **27**, 1596-1608 (2008).
5. Aphasizheva, I., Maslov, D., Wang, X., Huang, L. & Aphasizhev, R. Pentatricopeptide Repeat Proteins Stimulate mRNA Adenylation/Uridylation to Activate Mitochondrial Translation in Trypanosomes. *Molecular Cell* **42**, 106-117 (2011).
6. Zhang, L. et al. PPR polyadenylation factor defines mitochondrial mRNA identity and stability in trypanosomes. *EMBO J* **36**, 2435-2454 (2017).
7. Aphasizheva, I. et al. RNA binding and core complexes constitute the U-insertion/deletion editosome. *Mol. Cell Biol* **34**, 4329-4342 (2014).
8. Suematsu, T. et al. Antisense Transcripts Delimit Exonucleolytic Activity of the Mitochondrial 3' Processome to Generate Guide RNAs. *Mol Cell* **61**, 364-78 (2016).
9. Aphasizhev, R. & Aphasizheva, I. RNA Editing Uridylyltransferases of Trypanosomatids. *Methods in Enzymology* **424**, 51-67 (2007).
10. Kim, D.I., Jensen, S.C. & Roux, K.J. Identifying Protein-Protein Associations at the Nuclear Envelope with BioID. *Methods Mol Biol* **1411**, 133-46 (2016).
11. Fang, L. et al. Mapping the protein interaction network of the human COP9 signalosome complex using a label-free QTAX strategy. *Mol. Cell Proteomics* **11**, 138-147 (2012).
12. Aphasizheva, I., Zhang, L. & Aphasizhev, R. Investigating RNA editing factors from trypanosome mitochondria. *Methods* **107**, 23-33 (2016).
13. Zhang, J., Kobert, K., Flouri, T. & Stamatakis, A. PEAR: a fast and accurate Illumina Paired-End reAd mergeR. *Bioinformatics* **30**, 614-20 (2014).
