# Supplementary material for: Pentatricopeptide repeat poly(A) binding protein KPAF4 stabilizes mitochondrial mRNAs in Trypanosoma brucei
Source: Nat Commun. 2019 Jan 11;10:146. doi: 10.1038/s41467-018-08137-2 (PMC6329795; doi:10.1038/s41467-018-08137-2)
Supplement: Supplementary file 3 — Description of Additional Supplementary Files [file 41467_2018_8137_MOESM3_ESM.pdf]

Description of Additional Supplementary Files

File Name: Supplementary Data 1
Description: Raw mass spectrometry data for tandem affinity purified samples. Release 8.0 of T. brucei TREU927 database at http://tritrypdb.org was used for protein identification. Typical contaminations found in purified trypanosomal mitochondrial complexes have been removed. Raw data are provided in a separate sheet. Related to Figure 2.

File Name: Supplementary Data 2
Description: Raw mass spectrometry data for BioID experiments. Release 8.0 of T. brucei TREU927 database at http://tritrypdb.org was used for protein identification. Typical contaminations found in purified trypanosomal mitochondrial complexes have been removed. Raw data are provided in a separate sheet. Related to Figure 2.

File Name: Supplementary Data 3
Description: Sanger sequencing (96 well plate) of pre-edited RPS12 mRNA termini in KPAF4 RNAi cell line. RNA was isolated after 72 hours of RNAi induction. Thirteen representative sequences are listed.

File Name: Supplementary Data 4
Description: Mapping statistics for tails sequencing and CLAP experiments.

File Name: Supplementary Data 5
Description: Major components of KPAF4-WT and KPAF4-Mut complexes. Release 8.0 of T. brucei TREU927 database at http://tritrypdb.org was used for protein identification. Typical contaminations found in purified trypanosomal mitochondrial complexes have been removed. Raw data are provided in a separate sheet. Related to Figure 6

File Name: Supplementary Data 6
Description: DNA Oligonucleotides used in this study. Abbreviations: p, pre-edited; e, edited; fw, forward; rv, reverse.
