# Supplementary material for: Pentatricopeptide repeat poly(A) binding protein KPAF4 stabilizes mitochondrial mRNAs in Trypanosoma brucei
Source: Nat Commun. 2019 Jan 11;10:146. doi: 10.1038/s41467-018-08137-2 (PMC6329795; doi:10.1038/s41467-018-08137-2)
Supplement: Supplementary file 6 — Supplementary Data 3 [file 41467_2018_8137_MOESM6_ESM.docx]

**Supplementary Data 3.** Sanger sequencing (96 well plate) of pre-edited RPS12 mRNA termini in KPAF4 RNAi cell line. RNA was isolated after 72 hours of RNAi induction. Thirteen representative sequences are listed.

**>RPS12 encoded 3′ end reference**

AAGAGATTTTAGAATAAGATATGTTTTT

>KPAF4-C4

AAGAGATTTTAGAATAAGATATGTTTTTAAAAAAAAAAAAAAAAAAAAAAAAAAAAAAAAAAAAAAAAAAATTTTTTTTTTTT

>KPAF4-G6

AAAATATTTTAGAATAAGATATGTATTTTTAAAAAAAAAAAAAGTTAAAAAAAAAAATTTAAAAAAAAAATTTTTTTTTT

>KPAF4-D5

AAGTTAGATTTAGAT-ATAAGATATGTTTTTAAAAAAAAAAATATAAAAAAAATATTTTTTTTTT

>KPAF4-D7

AAGTTAGATTTAGATATAAGATATGTTTTTAAAAAAAAAAATATAAAAAAAATATTTTTTTTTT

>KPAF4-G5

AAGTTAGATTTAGATATAAGATATGTTAAAAAAAAAAAAAATAAAAAAAAAAAAAATTTTTTTTTTTATTTTT

>KPAF4-E12

AAGTTAGATTTAGATATAAGATATGTAAAAAAAAAAAAAAAAAAAAAAAAAAATTTTTTTTTTTTTT

>KPAF4-A4

AAGAGATTTTAGAATAAGATTTTTTTTAAAAAAAAAAATAAAAAAAAAAAAAAAAAAAAAATTTTTA

>KPAF4-F3

AAGTTAGATTTAGAATAAGATATGTTTTTAAAAAAAAAAAAAAAAAAAAAAAATTTTTTTTTTTTT

>KPAF4-C12

AAGTTAGATTTAGATATAAGATATGTTTTTAAAAAAAAAAATATAAAAAAAATATTTTTTTTTT

>KPAF4-H2

AAGAGATTTTAGAATAAGATATGTTTTTAAATAAAAAAAAAAAAAATATTTTTTTTTTTT

>KPAF4-D3

AAGTTAGATTTAGAT-ATAAGATATGTTTTTAAAAAAAAAAAAAAAAAATTTAATTT

>KPAF4-B2

AAGTTAGATTTAGATATAAGATATGTTTTTAAAAAAAATTTTTTTTT

>KPAF4-H3

AAGAGATTTTAGAATAAGATATGTTTTTAATTTAAATTTTAATAAAAAAAAAAAAAAAAATTATTT

>KPAF4-D7

AAGTTAGATTTAGAT-ATAAGATATGTTTTTAAAAAAAAAAAAAAAAAATTTAATTTTTT

>KPAF4-H8

AAGTTAGATTTAGATATAAGATATGTTTTTAAAAAAAAAAAAAAAAGAAAAAAAA

>KPAF4-G8

AAGAGATTTAGAATAAGATATGTTTTTAATATTTTTTTTATTTTTTATAAT

> KPAF4-F10

AAGTTAGATTTAGAT-ATAAGATATGTTTTTAAAAAAAAAAAAAAAAAAAAATT
