# Supplementary material for: Pentatricopeptide repeat poly(A) binding protein KPAF4 stabilizes mitochondrial mRNAs in Trypanosoma brucei
Source: Nat Commun. 2019 Jan 11;10:146. doi: 10.1038/s41467-018-08137-2 (PMC6329795; doi:10.1038/s41467-018-08137-2)
Supplement: Supplementary file 9 — Supplementary Data 6 [file 41467_2018_8137_MOESM9_ESM.docx]

**Supplementary Data 6.** DNA Oligonucleotides used in this study. Abbreviations: p, pre-edited; e, edited; fw, forward; rv, reverse.

| **Gene-specific primers for circular RT-PCR (cRT-PCR)** | |
| --- | --- |
| cRT 12S | CTATAAATATGTATCTAAATATAAAACAAC |
| cRT 9S | CAAAATTATAAATTATTATTAACTATTCC |
| cRT ND1 | ACGTTCACATAAACTAACATACC |
| cRT CO1 | ATAAATAAGGAGTAAATATAACCGATAAATCC |
| cRT CYBp/e | TCTATATAAACAACCTGACATTAAAAGAC |
| cRT CO3p | TCCTCCTCTGGCGGTGTC |
| cRT A6p | CAAAACCTTTCTCCTTCATTTCCTCTCC |
| cRT RPS12p | GGCTGGGCTCGAACGGCTC |
| cRT CO3e | ACAAAAACACCACTAACACCAAC |
| cRT A6e | AATACACAATAAAACATAACCTAGATCATACAC |
| cRT RPS12e | CGGACTCATATAATAACATAAAACAAATAAAAC |

| **cRT-PCR gene-specific primers for SMRT tail sequencing** | |
| --- | --- |
| SMRT 12S | TTTGAAATTATAAAAGGTTCGAGCAGG |
| SMRT 9S | GAAATTAAAAAGGTATTGTTGCCCAC |
| SMRT ND1 | TTATTTACAATGGGATTCATTAATTTTTC |
| SMRT CO1 | ATATTTTTTTTTGACAAGCCTTCC |
| SMRT CYBe | CAATATGAATGGAATTACAATACTGAG |
| SMRT CO3e | TTATGTATTTGTGTGTGTAATTTTATTGGT |
| SMRT A6e | ATATGATTTTGCAGTTGATAATGG |
| SMRT RPS12e | AGGGTGGTGGTTTTGTTGATTTACC |

| **cRT-PCR antisense gene-specific primers for SMRT tail sequencing** | |
| --- | --- |
| SMRT anti 12S | TATACAAAAAATCTTTCAAAAATAAACC |
| SMRT anti 9S | TATACAAAAAATCTTTCAAAAATAAACC |
| SMRT anti ND1 | CATAAGACAGATAAAACGAGTATAAATATAAC |
| SMRT anti CO1 | TAACCGATAAATCCACATAAAATAGC |
| SMRT anti CYBe | AACCTGACATTAAAAGACAACACA |
| SMRT anti CO3e | CCACTAACACCAACAAATATACAACGA |
| SMRT anti A6e | ACACAATAATACATACATAATAACAAACG |
| SMRT anti RPS12e | ATAAAACAAATAAAACAAAACGTAAACAACAAC |

| **cRT-PCR gene-specific primers for MiSeq tail sequencing** | |
| --- | --- |
| MiSeq 12S | GTTCAGAGTTCTACAGTCCGACGATCTTTGAAATTATAAAAGGTTCGAGCAGG |
| MiSeq 9S | GTTCAGAGTTCTACAGTCCGACGATCGAAATTAAAAAGGTATTGTTGCCCAC |
| MiSeq ND1 | GTTCAGAGTTCTACAGTCCGACGATCTTATTTACAATGGGATTCATTAATTTTTC |
| MiSeq CO1 | GTTCAGAGTTCTACAGTCCGACGATCATATTTTTTTTTGACAAGCCTTCC |
| MiSeq CYBp | GTTCAGAGTTCTACAGTCCGACGATCCAATATGAATGGAATTACAATACTGAG |
| MiSeq CO3p | GTTCAGAGTTCTACAGTCCGACGATCAAAGGGTTTAGTTGGAATGAAGAGG |
| MiSeq A6p | GTTCAGAGTTCTACAGTCCGACGATCATTGGGAATTGCCTTTGC |
| MiSeq RPS12p | GTTCAGAGTTCTACAGTCCGACGATCGAATAAAAGGGAGGCGGGGAG |

| **cRT-PCR antisense gene-specific primers for MiSeq tail sequencing** | |
| --- | --- |
| MiSeq anti 12S | GCCTTGGCACCCGAGAATTCCAAAATAATACAAATAATAAATTTACTACACGG |
| MiSeq anti 9S | GCCTTGGCACCCGAGAATTCCATATACAAAAAATCTTTCAAAAATAAACC |
| MiSeq anti ND1 | GCCTTGGCACCCGAGAATTCCACATAAGACAGATAAAACGAGTATAAATATAAC |
| MiSeq anti CO1 | GCCTTGGCACCCGAGAATTCCATAACCGATAAATCCACATAAAATAGC |
| MiSeq anti CYBp | GCCTTGGCACCCGAGAATTCCAGACCCTTTCTTTTTTCTCCGC |
| MiSeq anti CO3p | GCCTTGGCACCCGAGAATTCCACAAAAATCCCCTTCCAAAAAGC |
| MiSeq anti A6p | GCCTTGGCACCCGAGAATTCCATTCATTTCCTCTCCTGTCTCC |
| MiSeq anti RPS12p | GCCTTGGCACCCGAGAATTCCATCGAACGGCTCTTTCTCTCCAGTC |

| **Illumina MiSeq and NEBNext primers** | |
| --- | --- |
| Universal primer (fw) | AATGATACGGCGACCACCGAGATCTACACGTTCAGAGTTCTACAGTCCGA |
| Illumina index primer 1 (rv) | CAAGCAGAAGACGGCATACGAGATCGTGATGTGACTGGAGTTCCTTGGCACCCGAGAATTCCA |
| Illumina index primer 2 (rv) | CAAGCAGAAGACGGCATACGAGATACATCGGTGACTGGAGTTCCTTGGCACCCGAGAATTCCA |
| Illumina index primer 3 (rv) | CAAGCAGAAGACGGCATACGAGATGCCTAAGTGACTGGAGTTCCTTGGCACCCGAGAATTCCA |
| Illumina index primer 4 (rv) | CAAGCAGAAGACGGCATACGAGATTGGTCAGTGACTGGAGTTCCTTGGCACCCGAGAATTCCA |
| Illumina index primer 5 (rv) | CAAGCAGAAGACGGCATACGAGATCACTGTGTGACTGGAGTTCCTTGGCACCCGAGAATTCCA |
| Illumina index primer 6 (rv) | CAAGCAGAAGACGGCATACGAGATATTGGCGTGACTGGAGTTCCTTGGCACCCGAGAATTCCA |
| Illumina index primer 7 (rv) | CAAGCAGAAGACGGCATACGAGATGATCTGGTGACTGGAGTTCCTTGGCACCCGAGAATTCCA |
| Illumina index primer 8 (rv) | CAAGCAGAAGACGGCATACGAGATTCAAGTGTGACTGGAGTTCCTTGGCACCCGAGAATTCCA |
| Illumina index primer 9 (rv) | CAAGCAGAAGACGGCATACGAGATCTGATCGTGACTGGAGTTCCTTGGCACCCGAGAATTCCA |
| Illumina index primer 10 (rv) | CAAGCAGAAGACGGCATACGAGATAAGCTAGTGACTGGAGTTCCTTGGCACCCGAGAATTCCA |
| Illumina index primer 11 (rv) | CAAGCAGAAGACGGCATACGAGATGTAGCCGTGACTGGAGTTCCTTGGCACCCGAGAATTCCA |
| Illumina index primer 12 (rv) | CAAGCAGAAGACGGCATACGAGATTACAAGGTGACTGGAGTTCCTTGGCACCCGAGAATTCCA |
| Illumina index primer 13 (rv) | CAAGCAGAAGACGGCATACGAGATTTGACTGTGACTGGAGTTCCTTGGCACCCGAGAATTCCA |
| Illumina index primer 14 (rv) | CAAGCAGAAGACGGCATACGAGATGGAACTGTGACTGGAGTTCCTTGGCACCCGAGAATTCCA |
| Illumina index primer 15 (rv) | CAAGCAGAAGACGGCATACGAGATTGACATGTGACTGGAGTTCCTTGGCACCCGAGAATTCCA |
| Illumina index primer 16 (rv) | CAAGCAGAAGACGGCATACGAGATGGACGGGTGACTGGAGTTCCTTGGCACCCGAGAATTCCA |
| NEBNext Adaptor for Illumina | 5 ́-Phos-GATCGGAAGAGCACACGTCTGAACTCCAGTC/ideoxyU/ACACTCTTTCCCTACACGACGCTCTTCCGATCT |
| NEBNext Universal PCR Primer for Illumina | AATGATACGGCGACCACCGAGATCTACACTCTTTCCCTACACGACGCTCTTCCGATCT |
| NEBNext Index 1 Primer for Illumina | CAAGCAGAAGACGGCATACGAGATCGTGATGTGACTGGAGTTCAGACGTGTGCTCTTCCGATCT |
| NEBNext Index 2 Primer for Illumina | CAAGCAGAAGACGGCATACGAGATACATCGGTGACTGGAGTTCAGACGTGTGCTCTTCCGATCT |

| **Northern blot primers** | |
| --- | --- |
| PCR probe RPS12p fw | CGACGGAGAGCTTCTTTTGAATA |
| PCR probe RPS12p rv | CCCCCCACCCAAATCTTT |
| PCR probe RPS12e fw | CGTATGTGATTTTTGTATGGTTGTTG |
| PCR probe RPS12e rv | ACACGTCGGTTACCGGAACT |
| PCR probe A6p fw | TTGCCTTTGCCAAACTTTTAGAAG |
| PCR probe A6p rv | ATTCTATAACTCCAAAATCACAACTTTCC |
| PCR probe A6e fw | CAAACCAACAAACAAATACAAATCAAAC |
| PCR probe A6e rv | GATTTATTTTGGTTGCGTTTGTTATTATG |
| Oligo probe CYBp | GACCCTTTCTTTTTTCTCCGC |
| Oligo probe CYBe | TGACATTAAAAGACAACACAAATTTCTAAATAATAAAAAAAATAATAAAAATCTACAACGAAACATATTTATATAAAATTTATAACC |
| Oligo probe 9S rRNA | ACGGCTGGCATCCATTTC |
| Oligo probe 12S rRNA | TGAACAATCAATCATGGTAATAAGTAGACGATG |
| PCR probe CO1 fw | TGCCTATAACTATGGGTGGGTTTACAAAC |
| PCR probe CO1 rv | ACTAAGCAACCAAATCCTCCAATAAACATTC |
| PCR probe ND1 fw | GGACTGCTTCTTGATGGATTACGTTTACC |
| PCR probe ND1 rv | AGATAATTCAGTAACAAGGCCAGCAACAAG |
| Oligo probe 5.8S | GGAAGCCAAGTCATCCATCGCGACACGTTGTGGGAGCCGTGG |
| Oligo probe 18S | TGGTAAAGTTCCCCGTGTTGA |
| Oligo probe gA6 | ATAATTATCATATCACTGTCAAAATCTGATTCGTTATCGGAGTTATAGTATAT |
| Oligo probe gCO3 | TAATTAAATCTTCTCATTGTCACTGTCTTATACTACGATTGAGTTTGTAT |
| Oligo probe tRNA^Met^ | GTGAGGCTCGAACTCACG |

| **Quantitative RT-PCR primers** | |
| --- | --- |
| 9S fw | ATTAGATTGTTTTGTTAATGCTATTAGATG |
| 9S rv | ACGGCTGGCATCCATTTC |
| 12S fw | GGGCAAGTCCTACTCTCCTTTACAAAG |
| 12S rv | TGAACAATCAATCATGGTAATAAGTAGACGATG |
| ND1 fw | GGACTGCTTCTTGATGGATTACGTTTACC |
| ND1 rv | AGATAATTCAGTAACAAGGCCAGCAACAAG |
| ND4 fw | CAATCTGACCATTCCATGTGTGACTACC |
| ND4 rv | TGCTATAAATACTAAACCCAACACAATTACACTATC |
| ND5 fw | TTTCTATATGTTTGTTAGTAGGATGTGCGTTC |
| ND5 rv | GCGTGTATTAATGCTGATACTGGGATAGG |
| MURF1 fw | GTTTACTACTTGCATGTCTCTTTCTTTG |
| MURF1 rv | AAAGCCAATACAAATACAAAGGTAACTTAG |
| CO1 fw | TGCCTATAACTATGGGTGGGTTTACAAAC |
| CO1 rv | ACTAAGCAACCAAATCCTCCAATAAACATTC |
| A6p fw | GAGAAGCAAGGAGGAGAA |
| A6p rv | GCAAAGGCAATTCCCAAT |
| A6e fw | TTGCCGCCATATTACAGT |
| A6e rv | TCTATAACTCCAATAACAAACCAAAT |
| RPS12p fw | CGACGGAGAGCTTCTTTTGAATA |
| RPS12p rv | CCCCCCACCCAAATCTTT |
| RPS12e fw | CGTATGTGATTTTTGTATGGTTGTTG |
| RPS12e rv | ACACGTCGGTTACCGGAACT |
| MURF2p fw | GATTTTAAGATTGGCTTTGATTGA |
| MURF2p/e rv | AATATAAAATCTAGATCAAACCATCACA |
| MURF2e fw | GATTTTAATGTTTGGTTGTTTTAATTTA |
| CYBp fw | ATATAAAAGCGGAGAAAAAAGAAAG |
| CYBp rv | CCCATATATTCTATATAAACAACCTGACA |
| CYBe fw | ATATAAATATGTTTCGTTGTAGATT |
| CYBe rv | CTAAACACACTCCACAAAT |
| ND3p fw | GAATGGGAGATGGGTTTTGG |
| ND3p rv | AACAAATCTCTTTACCCCCTTCAG |
| ND3e fw | CGTTGTTGTTTGTGGTTT |
| ND3e rv | ACAAATAATGGAATTTAACAATACA |
| ND7p fw | GCGGGCGGAGCATTATT |
| ND7p rv | GATCTACGGTCCCCTCTTTCC |
| ND7e fw | GCATCCCGCAGCACATG |
| ND7e rv | CTGTACCACGATGCAAATAACCTATAAT |
| CO3p fw | GGGAAACCAGATGAGATTG |
| CO3p rv | ACTACCTCTTCATTCCAACTA |
| CO3e fw | GAAACCAGATGAGATTGTTTGCA |
| CO3e rv | TTCATTCCAACTAAACCCTTTCC |
| CO2p/e fw | ATTACAGTGTAACCATGTATTGACATT |
| CO2p rv | TTCATTACACCTACCAGGTTCTCT |
| CO2e rv | ATTTCATTACACCTACCAGGTATACAA |
| β-tubulin fw | TTCCGCACCCTGAAACTGA |
| β-tubulin rv | TGACGCCGGACACAACAG |
| KPAF4 fw |  |
| KPAF4 rv |  |
