# Supplementary material for: Pentatricopeptide repeat poly(A) binding protein KPAF4 stabilizes mitochondrial mRNAs in Trypanosoma brucei
Source: Nat Commun. 2019 Jan 11;10:146. doi: 10.1038/s41467-018-08137-2 (PMC6329795; doi:10.1038/s41467-018-08137-2)
Supplement: Supplementary file 11 — Reporting Summary [file 41467_2018_8137_MOESM11_ESM.pdf]

nature research

Corresponding author(s): *Double-blind peer review submissions: write DBPR and your manuscript number here instead of author names.*

Last updated by author(s): *YYYY-MM-DD*

Reporting Summary

Nature Research wishes to improve the reproducibility of the work that we publish. This form provides structure for consistency and transparency in reporting. For further information on Nature Research policies, see Authors & Referees and the Editorial Policy Checklist.

Statistics

For all statistical analyses, confirm that the following items are present in the figure legend, table legend, main text, or Methods section.

n/a	Confirmed	
☐	☒	The exact sample size (*n*) for each experimental group/condition, given as a discrete number and unit of measurement
☒	☐	A statement on whether measurements were taken from distinct samples or whether the same sample was measured repeatedly
☒	☐	The statistical test(s) used AND whether they are one- or two-sided<br>*Only common tests should be described solely by name; describe more complex techniques in the Methods section.*
☐	☒	A description of all covariates tested
☒	☐	A description of any assumptions or corrections, such as tests of normality and adjustment for multiple comparisons
☒	☐	A full description of the statistical parameters including central tendency (e.g. means) or other basic estimates (e.g. regression coefficient) AND variation (e.g. standard deviation) or associated estimates of uncertainty (e.g. confidence intervals)
☒	☐	For null hypothesis testing, the test statistic (e.g. *F*, *t*, *r*) with confidence intervals, effect sizes, degrees of freedom and *P* value noted<br>*Give P values as exact values whenever suitable.*
☒	☐	For Bayesian analysis, information on the choice of priors and Markov chain Monte Carlo settings
☒	☐	For hierarchical and complex designs, identification of the appropriate level for tests and full reporting of outcomes
☒	☐	Estimates of effect sizes (e.g. Cohen's *d*, Pearson's *r*), indicating how they were calculated

Our web collection on statistics for biologists contains articles on many of the points above.

Software and code

Policy information about availability of computer code

Data collection

Provide a description of all commercial, open source and custom code used to collect the data in this study, specifying the version used OR state that no software was used.

Data analysis

Open source software:
1. Bowtie2 (v2.3.2): Bowtie 2 is an ultrafast and memory-efficient tool for aligning sequencing reads to long reference sequences.
2. BWA (v0.7.15): BWA is a software package for mapping low-divergent sequences against a large reference genome.
3. Cutadapt (v1.14): Cutadapt finds and removes adapter sequences, primers, poly-A tails and other types of unwanted sequence from your high-throughput sequencing reads.
4. PEAR (0.9.8): PEAR is an ultrafast, memory-efficient and highly accurate pair-end read merger.

Custom code:
1. baseCtByPos.pl: Code to generate base counts by read nucleotide position. It reads one-line fasta file as input, and outputs base counts for each position of the reads.
2. clean_tails.pl: Code to remove irrelevant reads in tail reads. It reads one-line fasta file as input, and outputs reads that contains G or C lower than a given percentage threshold.
3. fa_collapse_prefix.pl: Code to merge identical reads to unique reads with a count number. It reads one-line fasta file as input, and outputs unique reads with identifiers that compose of a given prefix and a count number.
4. fasta_count.pl: Code to count the total number of reads in a fasta file.
5. fq2fa_collapse_prefix.pl: Code to convert fastq file to fasta file, and merge identical reads to unique reads. It reads fastq file as input, and outputs unique reads with identifiers that compose of a given prefix and a count number.
6. fqPE_rename_split.pl: Code to split paired-end fastq format sequencing data to a given number of partitions. It reads two fastq files as input, and takes a third argument as the prefix of the output filename. The output is a given number of fastq files which are partitions of the original fastq files.
7. sam2maskedBaseFreq.pl: Code to count the number of reads mapped to as well as soft-masked at each position on the reference genome. It reads SAM files as input, and output a file where each line contains the position of the genome, read count mapped to the

position, and each of the base soft-masked at the position.

For manuscripts utilizing custom algorithms or software that are central to the research but not yet described in published literature, software must be made available to editors/reviewers. We strongly encourage code deposition in a community repository (e.g. GitHub). See the Nature Research guidelines for submitting code & software for further information.

Data

Policy information about availability of data

All manuscripts must include a data availability statement. This statement should provide the following information, where applicable:

- Accession codes, unique identifiers, or web links for publicly available datasets
- A list of figures that have associated raw data
- A description of any restrictions on data availability

All data generated or analyzed during this study are included in this article (and its Supplementary Information files). KPAF4 CLAP-Seq and tail sequencing data were deposited into the Sequence Read Archive (https://www.ncbi.nlm.nih.gov/sra) under accession number PRJNA477550. Sequence analysis scripts are available at www.tinyurl.com/y7x2txkh.

Field-specific reporting

Please select the one below that is the best fit for your research. If you are not sure, read the appropriate sections before making your selection.

☒ Life sciences ☐ Behavioural & social sciences ☐ Ecological, evolutionary & environmental sciences

For a reference copy of the document with all sections, see nature.com/documents/nr-reporting-summary-flat.pdf

Life sciences study design

All studies must disclose on these points even when the disclosure is negative.

Sample size	RNA and protein samples contained internal reference molecules. These were used as loading or normalization standards in replicate experiments, as indicated in the text. For northern and western blotting, tail sequencing and CLIP sequencing two replicas were performed in most cases. For qRT-PCR, four experiments were performed and standard deviation calculated.
Data exclusions	RNA-seq and CLIP-seq reads mapping to nuclear genome were excluded from analysis of mitochondrial transcripts.
Replication	Northern and western blotting, tail sequencing and CLIP sequencing experiments were considered reproducible if two replicates produced similar results.
Randomization	No randomization has been performed.
Blinding	Blinding is not a standard practice for the type of experiments described in the paper.

Reporting for specific materials, systems and methods

We require information from authors about some types of materials, experimental systems and methods used in many studies. Here, indicate whether each material, system or method listed is relevant to your study. If you are not sure if a list item applies to your research, read the appropriate section before selecting a response.

Materials & experimental systems

n/a	Involved in the study	
☐	☒	Antibodies
☐	☒	Eukaryotic cell lines
☐	☐	Palaeontology
☐	☐	Animals and other organisms
☐	☐	Human research participants
☐	☐	Clinical data

Methods

n/a	Involved in the study	
☒	☐	ChIP-seq
☒	☐	Flow cytometry
☒	☐	MRI-based neuroimaging

Antibodies

Antibodies used	All in-house produced antibodies used in this work have been validated in peer-reviewed publications.<br>KPAP1<br>Etheridge, R.D., Aphasizheva, I., Gershon, P.D. & Aphasizhev, R. 3' adenylation determines mRNA abundance and monitors completion of RNA editing in T. brucei mitochondria. EMBO J 27, 1596-1608 (2008).<br>RET1<br>Suematsu, T. et al. Antisense Transcripts Delimit Exonucleolytic Activity of the Mitochondrial 3' Processome to Generate Guide

RNAs. Mol Cell 61, 364-78 (2016).
KPAF3
Zhang, L. et al. PPR polyadenylation factor defines mitochondrial mRNA identity and stability in trypanosomes. EMBO J 36, 2435-2454 (2017).
GRBC1/2
Aphasizheva, I. & Aphasizhev, R. RET1-catalyzed Uridylylation Shapes the Mitochondrial Transcriptome in Trypanosoma brucei. Molecular and Cellular Biology 30, 1555-1567 (2010).
MERS1
Sement FM, S.T., Zhang L, Yu T, Huang L, Aphasizheva I, Aphasizhev R. Transcription initiation defines kinetoplast RNA boundaries. PNAS 115, epub Oct 17 (2018).
KPAF1
Aphasizheva, I., Maslov, D., Wang, X., Huang, L. & Aphasizhev, R. Pentatricopeptide Repeat Proteins Stimulate mRNA Adenylation/Uridylation to Activate Mitochondrial Translation in Trypanosomes. Molecular Cell 42, 106-117 (2011).

Commercial tag-specific antibody
Thermo Fisher Scientific

Validation

Describe the validation of each primary antibody for the species and application, noting any validation statements on the manufacturer's website, relevant citations, antibody profiles in online databases, or data provided in the manuscript.

Eukaryotic cell lines

Policy information about cell lines

Cell line source(s)	Trypanosoma brucei Plimmer and Bradford (ATCC® PRA-381™)<br>Trypanosoma brucei Lister 427 29-13 (TetR T7RNAP) transgenic procyclic form
Authentication	ATCC deposited cell line. Validated in Wirtz E, et al. A tightly regulated inducible expression system for conditional gene knock-outs and dominant-negative genetics in Trypanosoma brucei. Mol. Biochem. Parasitol. 99(1): 89-101, 1999. PubMed: 10215027
Mycoplasma contamination	trypanosomal cell line, not a subject to mycopalsma Mycoplasma contamination
Commonly misidentified lines<br>(See ICLAC register)	None

Palaeontology

Specimen provenance	N/A
Specimen deposition	N/A
Dating methods	N/A

☐ Tick this box to confirm that the raw and calibrated dates are available in the paper or in Supplementary Information.

Animals and other organisms

Policy information about studies involving animals; ARRIVE guidelines recommended for reporting animal research

Laboratory animals	*For laboratory animals, report species, strain, sex and age OR state that the study did not involve laboratory animals.*
Wild animals	*Provide details on animals observed in or captured in the field; report species, sex and age where possible. Describe how animals were caught and transported and what happened to captive animals after the study (if killed, explain why and describe method; if released, say where and when) OR state that the study did not involve wild animals.*
Field-collected samples	*For laboratory work with field-collected samples, describe all relevant parameters such as housing, maintenance, temperature, photoperiod and end-of-experiment protocol OR state that the study did not involve samples collected from the field.*
Ethics oversight	*Identify the organization(s) that approved or provided guidance on the study protocol, OR state that no ethical approval or guidance was required and explain why not.*

Note that full information on the approval of the study protocol must also be provided in the manuscript.

Human research participants

Policy information about studies involving human research participants

Population characteristics	*Describe the covariate-relevant population characteristics of the human research participants (e.g. age, gender, genotypic information, past and current diagnosis and treatment categories). If you filled out the behavioural & social sciences study design questions and have nothing to add here, write "See above."*

Recruitment	*Describe how participants were recruited. Outline any potential self-selection bias or other biases that may be present and how these are likely to impact results.*
Ethics oversight	*Identify the organization(s) that approved the study protocol.*

Note that full information on the approval of the study protocol must also be provided in the manuscript.

Clinical data

Policy information about clinical studies

All manuscripts should comply with the ICMJE guidelines for publication of clinical research and a completed CONSORT checklist must be included with all submissions.

Clinical trial registration	*Provide the trial registration number from ClinicalTrials.gov or an equivalent agency.*
Study protocol	*Note where the full trial protocol can be accessed OR if not available, explain why.*
Data collection	*Describe the settings and locales of data collection, noting the time periods of recruitment and data collection.*
Outcomes	*Describe how you pre-defined primary and secondary outcome measures and how you assessed these measures.*
